# Development of an endoplasmic reticulum stress-related signature with potential implications in prognosis and immunotherapy in head and neck squamous cell carcinoma

**DOI:** 10.1186/s13000-023-01338-4

**Published:** 2023-04-22

**Authors:** Xinlong Fan, Xiao Yang, Nan Guo, Xin Gao, Yuejiao Zhao

**Affiliations:** Second Ward of Head & Neck Surgery, Liaoning Cancer Hospital & Institute, Cancer Hospital of China Medical University, No.44 Xiaoheyan Road, Dadong District, 110042 Shenyang, Liaoning Province P R China

**Keywords:** Head and neck squamous cell carcinoma, Endoplasmic reticulum stress, Immunotherapy, Prognosis, Biomarker

## Abstract

**Background:**

Head and neck squamous cell carcinoma (HNSCC) is a multisite malignancy that responds well to immunotherapy. Despite the initial enthusiasm, the clinical benefits of immunotherapy in HNSCC patients are overall limited. Endoplasmic reticulum stress (ERS) has been indicated to play a key role in the process of anti-tumor immune response mediation. However, ERS-related biomarkers which can accurately predict prognosis and immunotherapy response in HNSCC are still lacking.

**Methods and results:**

In this study, we identify and validate an ERS-related signature comprises of six genes (ASNS, EXOSC6, BAK1, TPP1, EXOSC8, and TATDN2) that can predict the prognosis of HNSCC patients. GSEA analysis indicates that the ERS-related signature is significantly correlated with tumor immunity in HNSCC. Moreover, the infiltration of naive B cells and CD8 + T cells are significantly diminished in patients with high-risk scores compared to those with low-risk scores, while macrophages and activated mast cells are remarkably enhanced. Furthermore, the ERS-related signature also displays a tremendous potential for predicting immunotherapy response in HNSCC.

**Conclusions:**

Our study identifies an ERS-related signature that can predict the prognosis of HNSCC patients and highlights its potential value as a predictive biomarker of immunotherapy response, potentially enabling more precise and personalized immunotherapy response and paving the way for further investigation of the prognostic and therapeutic potentials of ERS.

**Supplementary Information:**

The online version contains supplementary material available at 10.1186/s13000-023-01338-4.

## Introduction

Head and neck cancer (HNC) is the seventh most common cancer worldwide, affecting about 453 000 deaths [1], of which head and neck squamous cell carcinoma (HNSCC) is the most common type. Despite rapid advances in treatment for HNC, the 5-year overall survival rate of patients remains at about 50% [2]. In addition, the classic clinical factors including lymph node metastasis and histological grade are not sufficient to predict the prognosis of patients, owing to the heterogeneity of molecular mechanisms and tumor behaviors related to HNSCC [3]. Therefore, identifying clinically relevant biomarkers for HNSCC can effectively improve the prognosis of patients. However, an accurate biomarker for the prognosis of HNSCC patients is still lacking in the clinic.

Immunotherapies eradicate cancerous cells by enhancing immune system activity [4]. Immune checkpoint inhibitors (ICIs) are an effective type of immunotherapies that block inhibitory immune checkpoint pathways to enhance anti-tumor immune activity [5]. In 2016, the US Food and Drug Administration (FDA) granted the first immunotherapeutic approval, for nivolumab and pembrolizumab, as anti-PD-1 immune checkpoint inhibitors, for the treatment of patients with recurrent HNSCC that is refractory to platinum-based regimens. The European Commission followed in 2017 with approval of nivolumab for the treatment of the same patient population, and shortly thereafter with the approval of pembrolizumab monotherapy for the treatment of recurrent or metastatic HNSCC in adults whose tumors express PD-L1 with a ≥ 50% tumor proportion score and have progressed on or after platinum-containing chemotherapy. Then in 2019, the FDA approved PD-1 inhibition as the first-line treatment for patients with metastatic or unresectable, recurrent HNSCC [6]. Despite the successful application of immunotherapy across a broad range of human cancers, the majority of patients have limited or no response to these therapies [7]. Therefore, excavating a predictive biomarker to assess the response to immunotherapies for defining patient benefit early is a desperate need. However, accurate biomarkers for predicting clinical outcomes and immunotherapy responses continue to be largely unexplored.

The Endoplasmic reticulum (ER) is an organelle widely present in eukaryotic cells that regulates protein synthesis, folding, and aggregation after synthesis. An aggregation of misfolded proteins in the lumen and an imbalance of Ca2 + in the cytoplasm caused by various factors can lead to ER dysfunction, which can induce a series of related protein expression and cell phenotype changes, a condition called endoplasmic reticulum stress (ERS) [8]. ERS has been proven to usually play an essential role in cell function and survival [9]. A previous study showed that ceramide synthase 1 could promote the aggressiveness of oral squamous cell carcinoma and chemotherapeutic drug resistance through ERS [10]. Furthermore, the expression of PERK-ATF4 has been indicated to be positively correlated with VEGF [11]. Thus, we can find that ERS may be associated with the prognosis of HNSCC and has enormous potential to predict it. However, there has been no report on ERS-related biomarkers for clinical outcomes and immunotherapy responses of HNSCC patients.

Therefore, in this study, we identify and validate an ERS-related signature for predicting the prognosis of HNSCC patients. The ERS-related signature showed a significant correlation with tumor immunity in Gene Set Enrichment Analysis (GSEA) analysis. Moreover, the infiltration of naive B cells and CD8 + T cells are significantly elevated in patients with low-risk scores compared to those with high-risk scores. By contrast, macrophages and activated mast cells were remarkably enhanced in high-risk groups compared to low-risk groups. Furthermore, we also find that the ERS-related signature can be a potential biomarker for immunotherapy response in HNSCC. This is pioneering research to identify an ERS-related signature that provides further insights for the prediction of prognosis and immunotherapy response of HNSCC patients. Moreover, a detailed analysis of the cellular immune response in HNSCC has the potential to enhance clinical and immunotherapy response prediction.

## Materials and methods

### Data and resources

Gene expression data and associated clinical characteristics of HNSCC patients were downloaded from the Cancer Genome Atlas (TCGA, http://cancergenome.nih.gov/). We downloaded RNA-Seq data expressed as transcripts per million (TPM) from TCGA database. R package ‘edgeR’ was utilized to normalize and process the data by using R version 4.0.4 software. This cohort has 487 HNSCC patients with the corresponding gene expression data and clinical information (Table 1).

**Table 1:** Clinical characteristics of HNSCC patients in TCGA database

Clinical characteristics	N	%
**Age(year)**		
**< 60**	215	44.15
**≥ 60**	272	55.15
**Stage**		
**I+II**	104	21.36
**III+IV**	383	78.64
**Pathological T**		
**T1 + T2**	172	35.32
**T3 + T4**	305	62.63
**TX**	10	2.05
**Pathological N**		
**N0**	236	48.46
**N1 + N2 + N3**	233	47.84
**NX**	18	3.70
**Pathological M**		
**M0**	463	95.07
**M1**	5	1.03
**MX**	19	3.90
**Grade**		
**G1 + G2**	352	72.28
**G3 + G4**	119	24.44
**GX**	16	3.28
**Status**		
**Alive**	277	56.88
**Death**	210	43.12
**Cancer status**		
**Tumor free**	309	69.44
**With tumor**	136	30.56
**Alcohol history**		
**No**	151	31.72
**Yes**	325	68.27
**New event**		
**No**	321	65.91
**Yes**	166	34.09

Microarray datasets including gene expression profiles and corresponding clinical information data of GSE65858 were downloaded from the Gene Expression Omnibus database (GEO, https://www.ncbi.nlm.nih.gov/geo/). GSE65858 was conducted by GPL10558 ( Illumina HumanHT-12 V4.0 expression beadchip), including 270 HNSCC samples which were involved in this work as a validating set.

### Construction and confirmation of a prognostic signature

In view of the essential role of ERS in cell function and survival [9], we dedicated to study ERS-related genes in HNSCC. An ERS-related gene set was defined based on Molecular Signatures Database (MSigDB, https://www.gsea-msigdb.org/gsea/msigdb/index.jsp), including GO:0036500 [12], GO:0036498 [12] and R-HSA-380,994 gene sets. These gene lists were displayed in Table 2. Then, to exploit genes associated with overall survival of HNSCC patients, we analyzed these genes by using univariate Cox regression analysis. Genes with P < 0.05 were further applied to multivariate Cox regression analysis. Thus, a prognostic signature of overall survival which was correlated with ERS was developed for HNSCC.

**Table 2:** The list of gene sets

ERS-related genes	ACADVL, ADD1, AGR2, ARFGAP1, ATP6V0D1, BAK1, BAX, BCL2L11, BFAR, COPS5, CTDSP2, CUL7, CXXC1, DAB2IP, DCTN1, DDRGK1, DDX11, DNAJB11, DNAJB9, DNAJC3, EDEM1, ERN1, ERN2, EXTL1, EXTL2, EXTL3, FICD, FKBP14, GET3, GFPT1, GOSR2, GSK3A, HDGF, HSPA5, HYOU1, KDELR3, KLHDC3, LMNA, MYDGF, PARP16, PDIA5, PDIA6, PLA2G4B, PPP2R5B, PREB, PTPN1, SEC31A, SERP1, SHC1, SRPRA, SRPRB, SSR1, SULT1A3, SULT1A4, SYVN1, TATDN2, TLN1, TMEM33, TPP1, TSPYL2, UFL1, VAPB, WFS1, WIPI1, XBP1, YIF1A, ZBTB17, ASNS, ATF3, ATF4, ATF6, CCL2, CEBPB, CEBPG, CXCL8, DCP2, DDIT3, DIS3, EXOSC1, EXOSC2, EXOSC3, EXOSC4, EXOSC5, EXOSC6, EXOSC7, EXOSC8, EXOSC9, HERPUD1, IGFBP1, KHSRP, NFYA, NFYB, NFYC, PARN, ATF6B, CALR, HSP90B1, MBTPS1, MBTPS2
Check-point genes	IDO1, LAG3, CTLA4, TNFRSF9, ICOS, CD80, PDCD1LG2, TIGIT, CD70, TNFSF9, ICOSLG, KIR3DL1, CD86, PDCD1, LAIR1, TNFRSF8, TNFSF15, TNFRSF14, IDO2, CD276, CD40, TNFRSF4, TNFSF14, HHLA2, CD244, CD274, HAVCR2, CD27, BTLA, LGALS9, TMIGD2, CD28, CD48, TNFRSF25, CD40LG, ADORA2A, VTCN1, CD160, CD44, TNFSF18, TNFRSF18, BTNL2, C10orf54, CD200R1, TNFSF4, CD200, NRP1

Next, a risk score was calculated for HNSCC patients according to expression levels of the genes (expi) and coefficients of multivariate Cox regression analysis (bi). Then, patients were split into high-risk and low-risk groups in the TCGA and GEO cohorts by median risk score values, respectively. The formula used was as follows:

$$\text{R}\text{i}\text{s}\text{k}\text{s}\text{c}\text{o}\text{r}\text{e}=\sum _{i=1}^{n}expi*bi$$

### Evaluation of endoplasmic reticulum stress score and check-point score

ERS score and check-point score were calculated based on the single-sample gene-set enrichment analysis (ssGSEA) [13] using the ERS-related gene sets and check-point gene sets (Table 2) to quantify the expression levels of these genes for HNSCC patients. We estimated the ERS score and check-point score between low- and high- risk groups of HNSCC.

### Survival analysis

We compared the overall survival (OS), recurrence-free survival (RFS), disease-free survival (DSS) and progression-free survival (PFS) of HNSCC patients in low- and high- risk groups. Kaplan-Meier (K-M) curves were carried out to compare the difference in prognosis. *P* values from Log-rank tests were calculated, and less than 0.05 was considered statistically significant.

### Gene set enrichment analysis

To explore the potential biological functions of the ERS-related genes between low- and high- risk groups in HNSCC, we conducted Gene Set Enrichment Analysis (GSEA) based on the curated gene sets c5.go.bp.v7.4.symbols. Normalized *P*-value < 0.05 was considered statistically significant.

### Evaluation of tumor-infiltrating immune cells

Normalized gene expression data were used to estimate the relative proportions of the infiltrating immune cells using the CIBERSORT algorithm [14]. Gene expression datasets were prepared using standard annotation files and data uploaded to the CIBERSORT web portal (https://cibersort.stanford.edu), with the algorithm run using the default signature matrix at 1,000 permutations. Here, we applied the original CIBERSORT gene signature file LM22 and analyzed gene profiles of HNSCC patients from TCGA database grouped by risk score. Subsequently, we selected 410 samples (consisted of 201 low-risk samples and 209 high-risk samples) which met the criteria of CIBERSORT *P* value < 0.05.

### Statistical analysis

The expression profiles of mRNAs from TCGA and GEO database were shown as raw data and each mRNA was normalized by log2 transformation for further analysis. We plotted ROC curves using the R package ‘survivalROC’, and the area under the ROC curve (AUC) was used to assess the prognostic performance of the ERS-related signature. Statistical analysis was performed by using GraphPad Prism version 7.0 or SPSS version 19.0 software package. Significant difference between two various groups was using Mann–Whitney test. Chi-squared test was applied to calculate the difference of clinicopathologic factors between low- and high- risk groups in HNSCC samples from TCGA dataset. *P* < 0.05 was considered statistically significant. We calculated the correlation between two variables using the Spearman method. The threshold of *P* < 0.05 (Spearman’s correlation test) indicates the significance of correlation. A two-tailed *P* < 0.05 was considered statistically significant.

## Results

### The activity of ERS is significantly different between tumor and normal samples in HNSCC

**Fig. 1:**
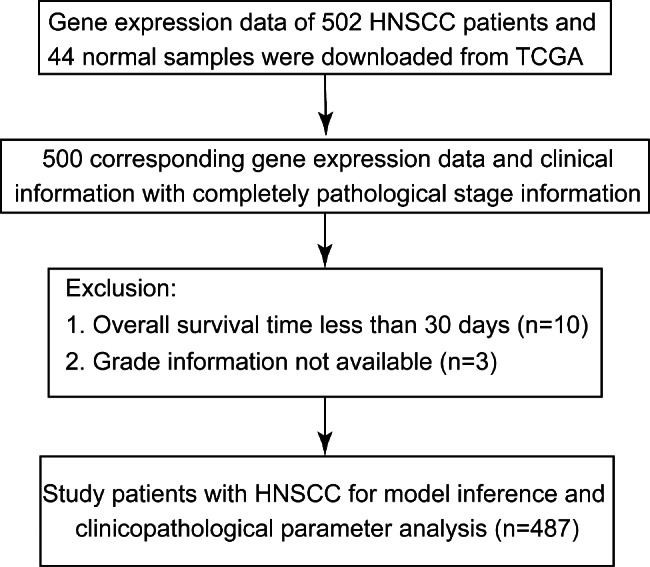
The patient flow diagram

**Fig. 2:**
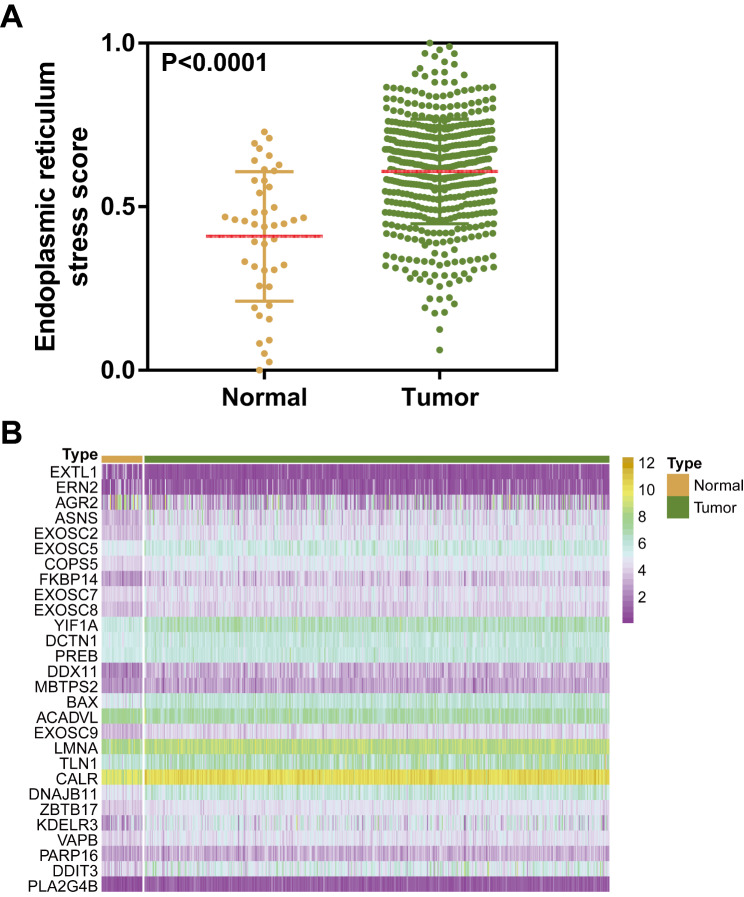
Activity of ERS is significantly different between tumor and normal samples in HNSCC. (**A**) Significant difference of ERS scores between normal and tumor samples in HNSCC. P-values are based on the Mann–Whitney test. (**B**) Heat map showing the expression levels of the 28 ERS-related genes differently expressed between normal and tumor samples in HNSCC from the TCGA.

In this study, the datasets of 502 HNSCC samples and 44 normal samples were obtained from the TCGA database. Two samples without a pathological stage, three samples without tumor grade information, and ten samples with OS less than 30 days were excluded (Fig. 1). To estimate the ERS activity of normal and tumor samples, we employed the ssGSEA algorithm using a list of ERS-related genes. The results showed that there was a significant difference in ERS activity between tumor and control samples in HNSCC (P < 0.0001, Fig. 2A). Moreover, tumor samples displayed higher ERS activity compared to normal samples. Then, we further analyzed the expression levels of 98 ERS-related genes in normal and tumor samples and found that there were 28 genes differently expressed between normal and tumor samples (P < 0.05, Fig. 2B; Table 3). These results suggest that ERS may be associated with the prognosis of HNSCC.

**Table 3:** The difference of the expression levels of 98 ERS-related genes between normal and tumor samples

Gene	F	Sig.	t
EXTL1	206.4991	6.61E-40	8.527505
ERN2	57.63663	1.39E-13	4.229211
AGR2	34.11768	8.95E-09	3.008987
ASNS	22.37632	2.86E-06	-7.36346
EXOSC2	14.43803	0.000161	-10.301
EXOSC5	13.56296	0.000254	-6.68298
COPS5	11.267	0.000844	-9.21954
FKBP14	10.47791	0.001282	-9.31252
EXOSC7	10.3245	0.001391	-4.44995
EXOSC8	10.08104	0.001583	-7.10474
YIF1A	8.614596	0.003476	-10.7409
DCTN1	7.942363	0.005005	-6.27158
PREB	6.80378	0.009347	-5.40872
DDX11	6.671145	0.01006	-10.5792
MBTPS2	6.455352	0.01134	-4.76203
BAX	6.293956	0.012406	-12.9178
ACADVL	6.286436	0.012458	5.685526
EXOSC9	6.209892	0.013002	-9.35448
LMNA	6.02593	0.01441	-6.60223
TLN1	5.935988	0.015156	-4.26637
CALR	4.945716	0.026567	-13.6833
DNAJB11	4.72582	0.030145	-12.7162
ZBTB17	4.717604	0.030288	-10.4849
KDELR3	4.685712	0.03085	-8.57213
VAPB	4.466379	0.035025	-5.47148
PARP16	4.282694	0.038976	-3.28037
DDIT3	4.050781	0.044645	-7.9171
PLA2G4B	3.964372	0.046974	-5.0994

### Identification of an ERS-related prognostic signature for HNSCC patients

**Fig. 3:**
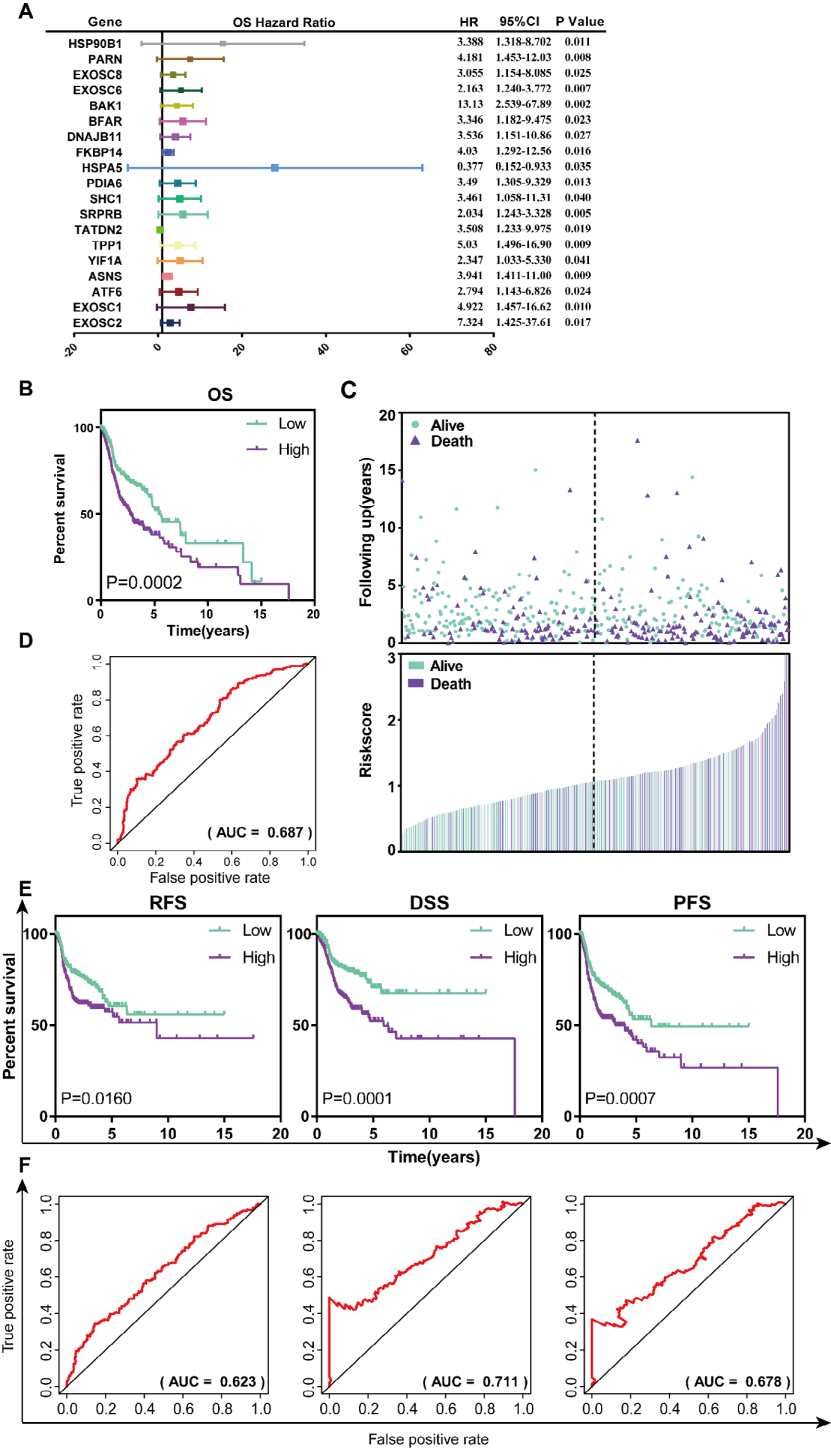
Construction of the ERS-related prognostic signature for OS of HNSCC in the TCGA dataset. (**A**) 19 genes related to OS of HNSCC were obtained by utilizing univariate Cox regression. (**B**) Kaplan–Meier curves of OS of low- and high- risk groups stratified by the risk score in HNSCC. (**C**) The distributions of risk scores and patient survival status for HNSCC. (**D**) ROC curve for the 5-year OS prediction by the ERS-related signature. (**E**) Kaplan–Meier curves of RFS, DSS and PFS of low- and high- risk groups stratified by the risk score in HNSCC, respectively. (**F**) ROC curves for the 5-year RFS, DSS and PFS prediction by the ERS-related signature, respectively

To exploit the prognosis-related genes from the 98 ERS-related genes in HNSCC, we first utilized univariate Cox regression analysis. Thus, 19 genes related to OS of HNSCC were obtained (P < 0.05, Fig. 3A; Table 4). Finally, as a result of multivariate Cox regression analysis, a six-mRNA (ASNS, EXOSC6, BAK1, TPP1, EXOSC8, and TATDN2) prognostic model was developed to predict the prognosis of HNSCC. Among these six genes, the expression of ASNS and EXOSC8 has a marked difference between normal and tumor samples in HNSCC (P < 0.05, Figure S1). Hence, based on these six genes, the HNSCC risk score system was established as follows: risk score= (0.6624 × expression value of ASNS) + (1.2499× expression value of EXOSC6) + (0.9704 × expression value of BAK1) + (1.1852 × expression value of TPP1) + (0.7885 × expression value of EXOSC8) + (-2.0666 × expression value of TATDN2).

**Table 4:** The list of 19 genes related to OS of HNSCC in univariate Cox regression analysis

Gene	HR	z	pvalue	Gene
HSPA5	13.13133	3.071866	0.002127	HSPA5
ASNS	2.034367	2.82672	0.004703	ASNS
FKBP14	2.1636	2.720435	0.00652	FKBP14
BFAR	4.181218	2.653028	0.007977	BFAR
EXOSC6	3.941341	2.618536	0.008831	EXOSC6
EXOSC1	5.030502	2.612301	0.008994	EXOSC1
PARN	4.922551	2.566099	0.010285	PARN
BAK1	3.388038	2.535093	0.011242	BAK1
TPP1	3.490236	2.491723	0.012713	TPP1
SRPRB	4.030575	2.40236	0.01629	SRPRB
HSP90B1	7.324101	2.384999	0.017079	HSP90B1
ATF6	3.50853	2.354241	0.018561	ATF6
PDIA6	3.346957	2.275343	0.022885	PDIA6
EXOSC8	2.794459	2.255136	0.024125	EXOSC8
DNAJB11	3.05514	2.249003	0.024512	DNAJB11
SHC1	3.536891	2.206326	0.027361	SHC1
TATDN2	0.377418	-2.1082	0.035014	TATDN2
YIF1A	3.461627	2.054721	0.039906	YIF1A

According to this formula, each patient has endowed a risk score. Then, 500 patients in the TCGA dataset were classified into high-risk and low-risk groups by the cut-off value of 1.058 as the median value of the risk score. Kaplan-Meier (K-M) curves showed that patients in high-risk groups tended to have poorer clinical outcomes compared to those in low-risk groups (Log-rank P = 0.0002, Fig. 3B). The distribution of gene risk scores and patients’ survival status of 500 HNSCC patients showed that patients’ mortality rate was augmented in the high-risk group compared to those in the low-risk group (Fig. 3C). Furthermore, the AUC value of 5-year OS was 0.687, which illustrated a medium accuracy of the ERS-related signature in OS prediction in HNSCC (Fig. 3D).

Given the predictive value of the signature for OS, we are curious whether the signature can also predict RFS, DFS, and PFS in HNSCC. Surprisingly, K-M curves illustrated that patients in high-risk groups had significantly shorter RFS, DFS, and PFS when compared with those in low-risk groups (RFS: Log-rank P = 0.0160, DFS: Log-rank P = 0.0001, PFS: Log-rank P = 0.0007, Fig. 3E). In addition, AUC values of 5-year RFS, DFS, and PFS were 0.623, 0.711, and 0.678, respectively, implying medium prediction performance of our signature for RFS, DFS, and PFS of HNSCC patients (Fig. 3F).

### The correlation of the ERS-related signature with clinical characteristics of HNSCC patients

**Fig. 4:**
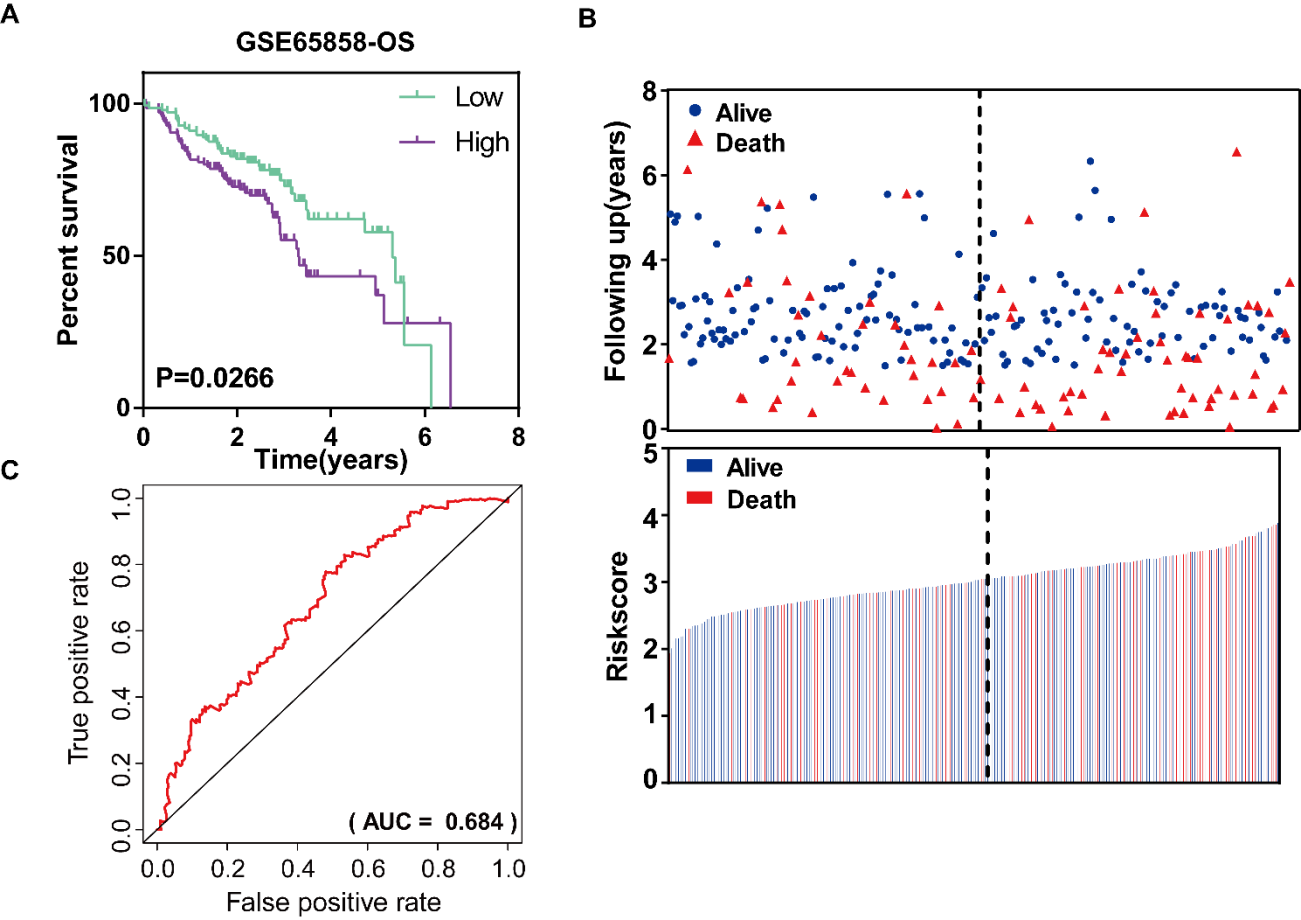
The relationship of the ERS-related signature with clinical characteristics of HNSCC patients. (**A**) The strip chart of risk score and clinical characteristics for patients with HNSCC in TCGA datatset. (**B**) Pie charts showing the Chi-squared test of clinicopathologic factors for low- and high- risk groups in HNSCC samples from TCGA dataset

Correlation analysis indicated that survival status, T, cancer status, perineural invasion present, and treatment response were significantly associated with the risk score based on the ERS-related signature (P < 0.05, Fig. 4A; Table 5). The strip chart showed that the risk of patient mortality rate elevated, while the T staging gradually rose and the treatment response rate markedly decreased as the risk score ascended (Fig. 3A). The chi-squared test demonstrated that the high-risk group significantly tended to high probability of death (P < 0.0001, Fig. 4B), older (P < 0.0001, Fig. 3B), survival with tumor (P = 0.0404, Fig. 4B) and no response to treatment (P = 0.0014, Fig. 4B) compared to the low-risk group of HNSCC. These results suggested that highly malignant HNSCC were associated with high-risk scores, and our risk score system based on the ERS-related signature had tremendous potential to predict prognosis for HNSCC patients.

**Table 5:** The correlation of the ERS-related signature with clinical characteristics of HNSCC.

Riskscore	R	P-value
**Survival status**	0.25990694	3.66E-09
**Sex**	0.009203876	0.837342
**Age**	0.068091548	0.128378
**T**	0.090	0.043778
** N**	-0.072036059	0.107653
**M**	0.056610167	0.206342
**Stage**	-0.007970425	0.858895
**Grade**	0.001420781	0.974719
**Cancer_Status**	0.207	3.1E-06
**Alcohol_history_documented**	0.053185789	0.235175
**Ethnicity**	0.023258122	0.603875
**Treatment_response**	-0.119	0.007556
**Lymphnode_neck_dissection**	0.086962586	0.052209
**Perineural_invasion_present**	0.126	0.00483

### The ERS-related signature is robust for predicting OS of HNSCC patients

**Fig. 5:**
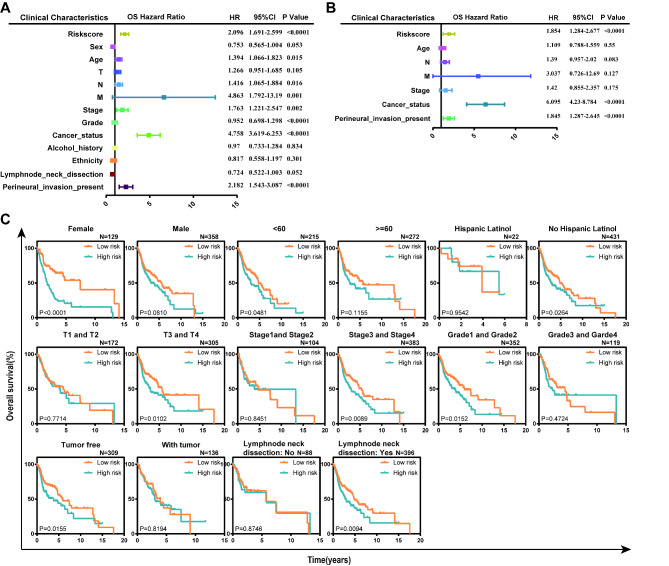
Validation of the ERS-related prognostic signature in GEO dataset. (**A**) Kaplan–Meier curves of OS of low- and high- risk groups stratified by the risk scores in HNSCC in GEO dataset. (**C**) The distributions of risk scores and patient survival status for HNSCC in TCGA dataset. (**D**) ROC curve for the 5-year OS prediction by the ERS-related signature in GEO dataset

To validate the predictive ability of the ERS-related signature, we applied the signature to the GSE65858 dataset. In this set, we used the same risk score model to calculate each patient’s risk score. Then, 270 patients were divided into high- and low-risk groups using the median of the risk scores value of 3.053. K-M curves implied that a higher risk score was associated with poorer prognosis in HNSCC, which was consistent with our previous findings (P = 0.0266, Fig. 5A). Moreover, the distributions of risk scores and patients’ survival status of 270 HNSCC patients showed that patients’ mortality rate increased as the risk score was elevated in HNSCC patients (Fig. 5B). The AUC value of 5-year OS was 0.684, implying a medium power of the signature in OS prediction of HNSCC (Fig. 5C).

### The ERS-related signature is an independently prognostic biomarker for HNSCC patients

**Fig. 6:**
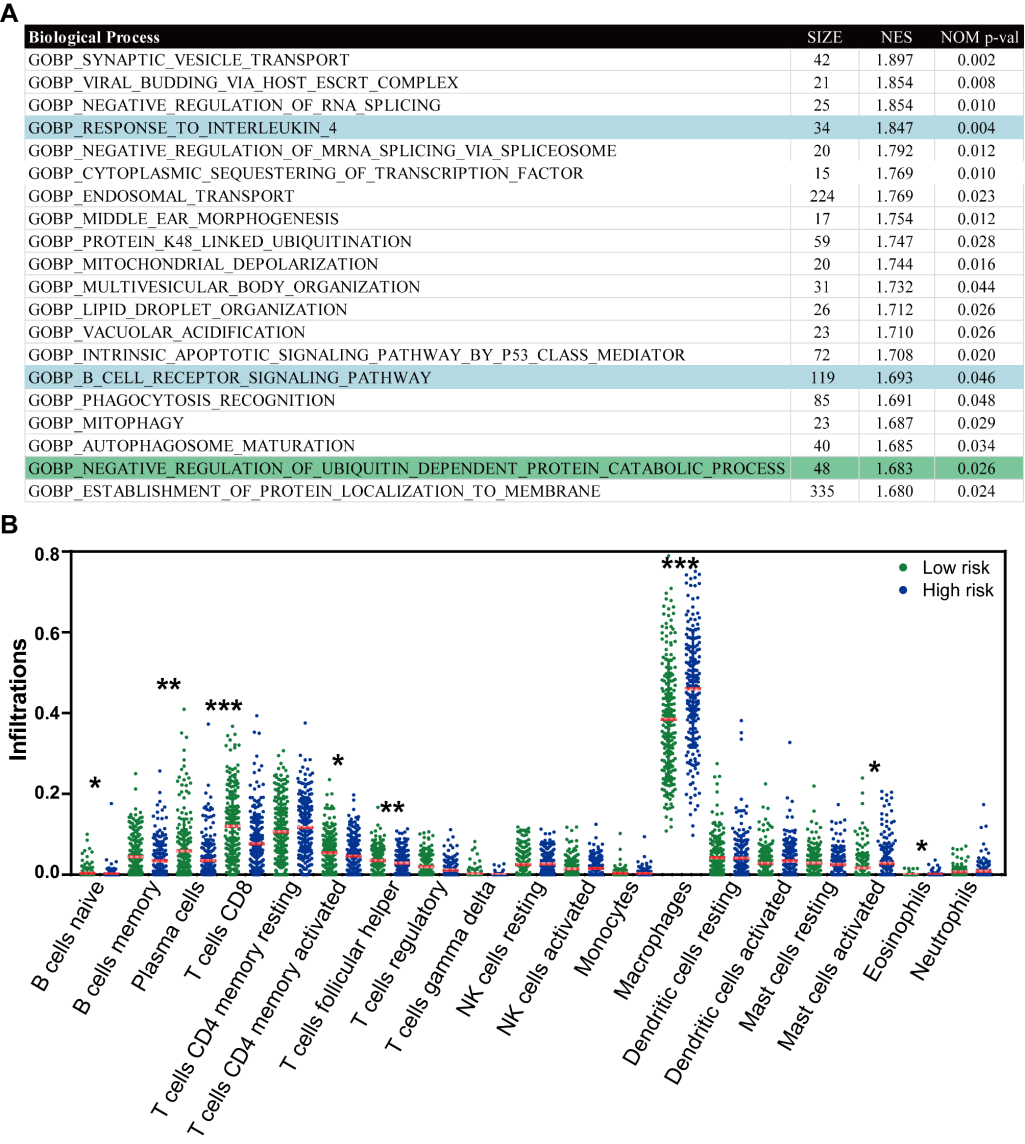
The ERS-related signature is an independent prognostic factor in HNSCC patients. Univariate (**A**) and multivariate (**B**) Cox regression of prognosis factor for OS of HNSCC patients. (**C**) Kaplan–Meier analysis of OS for HNSCC patients stratified by sex, age, ethnicity, T, stage, grade, cancer status and lymphnode neck dissection status

To investigate the independence of the prognostic signature, Cox regression analysis was carried out combined clinical characteristics with our ERS-related signature in the TCGA dataset. Firstly, univariate Cox regression analysis indicated that the risk score, age, N, M, stage, grade, cancer status, and perineural invasion present were prognostic factors for OS of HNSCC patients (P < 0.05, Fig. 6A). Moreover, multivariate Cox regression analysis showed that the ERS-related signature, cancer status, and perineural invasion present were independent predictors for OS, after adjusting to other four clinical factors (P < 0.05, Fig. 6B). Taken together, the above results suggest that the ERS-related signature is an independent adverse prognostic factor for OS of HNSCC patients, which relates to poor clinical outcomes.

Furthermore, stratified analyses based on these clinical characteristics were carried out to identify suitable patient groups for the prediction of the risk score system. The results showed that the risk score remained an independent prognostic factor for the subgroups of female, younger than 61, not Hispanic or Latino, T3 and T4, stage III and IV, grade 1 and 2, tumor-free and lymphnode neck dissection (P < 0.05, Fig. 6C). Moreover, the results of stratification analysis showed that high-risk patients in each stratum of those clinical parameters had significantly shorter OS than low-risk patients.

### The ERS-related signature is associated with tumor immunity in HNSCC

**Fig. 7:**
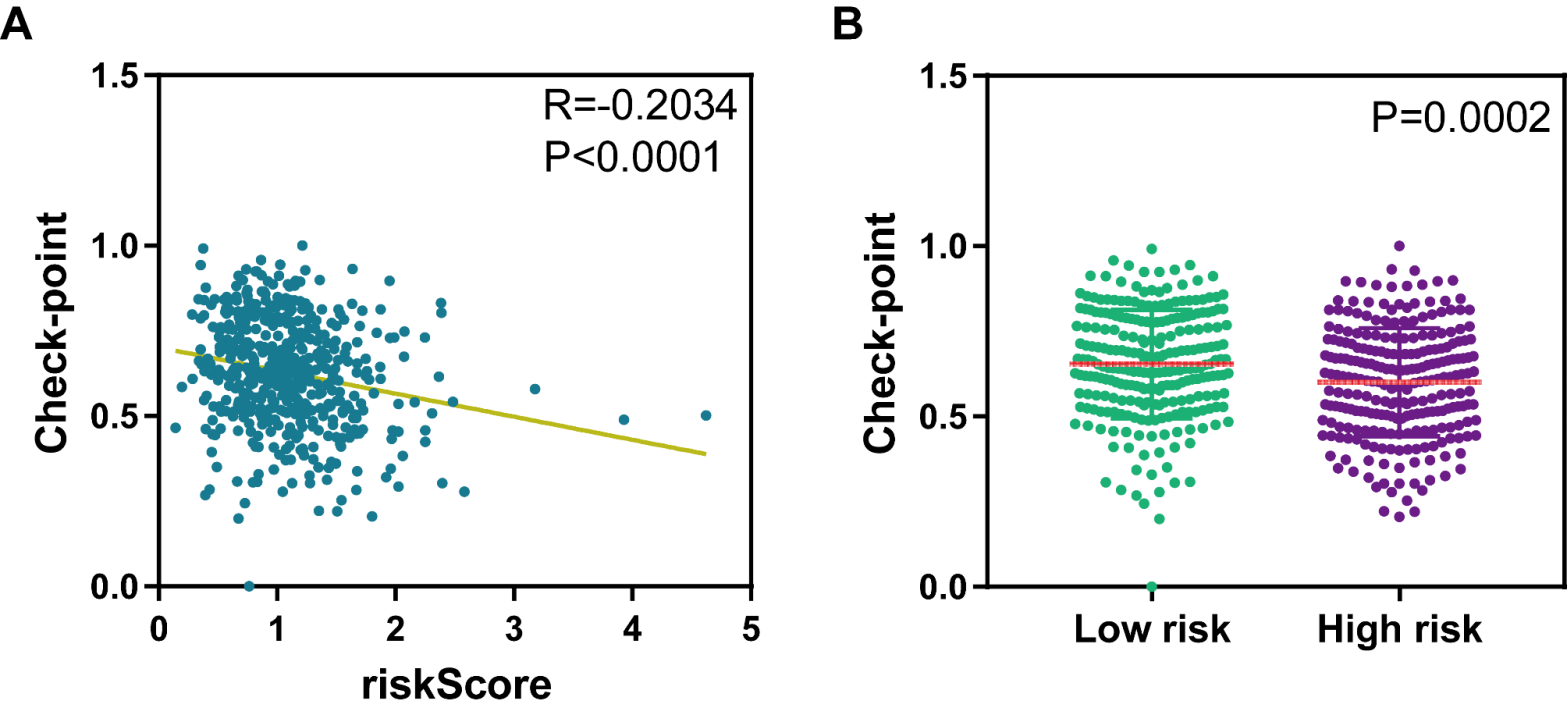
The ERS-related signature shows strongly association with immune surveillance against cancer cells in HNSCC. (**A**) The biological functions of the ERS-related signature in HNSCC using GSEA analysis based on the curated gene sets c5.go.bp.v7.4.symbols. (**B**) The difference of immune cell infiltration between low- and high- risk groups based on the ERS-related signature in HNSCC using CIBERSORT algorithm. P-values are based on the Mann–Whitney test. * P < 0.05, **P < 0.01, ***P < 0.001

To illustrate the potential biological functions of the ERS-related signature in HNSCC, we conducted a GSEA analysis based on the curated gene sets c5.go.bp.v7.4.symbols. We found that negative regulation of ubiquitin-dependent protein catabolic process was upregulated in low-risk score groups, which validated that our signature was ERS-associated (Fig. 7A). Moreover, some immune-associated pathways that upregulated in low-risk score groups in HNSCC, such as response to interleukin 4 and B cell receptor signaling pathway, imply our ERS-associated signature might be associated with immune surveillance against cancer cells.

A large body of research has proved that ERS usually affected many physiological processes, including immune function [15]. Our results also show that the ERS-related signature is associated with immune surveillance against cancer cells in HNSCC. Therefore, to further illustrate the potential mechanism of the correlation between the ERS-related signature and immune surveillance against cancer cells, we applied the CIBERSORT algorithm to the TCGA dataset. Excluding samples which CIBERSORT P-value > 0.05, 410 samples from the TCGA dataset were enrolled, including 201 low-risk samples and 209 high-risk samples. The analysis of immune cells infiltrations between low-risk and high-risk groups showed that the infiltrations of naive B cells, CD8 + T cells, follicular helper T cells, and plasma cells were significantly higher in low-risk groups than in high-risk groups (P < 0.05, Fig. 7B). However, macrophages and activated mast cells were remarkably enhanced in high-risk groups compared to those in low-risk groups (P < 0.05, Fig. 7B).

### The ERS-related signature has the potential to predict immunotherapy response

**Fig. 8:**
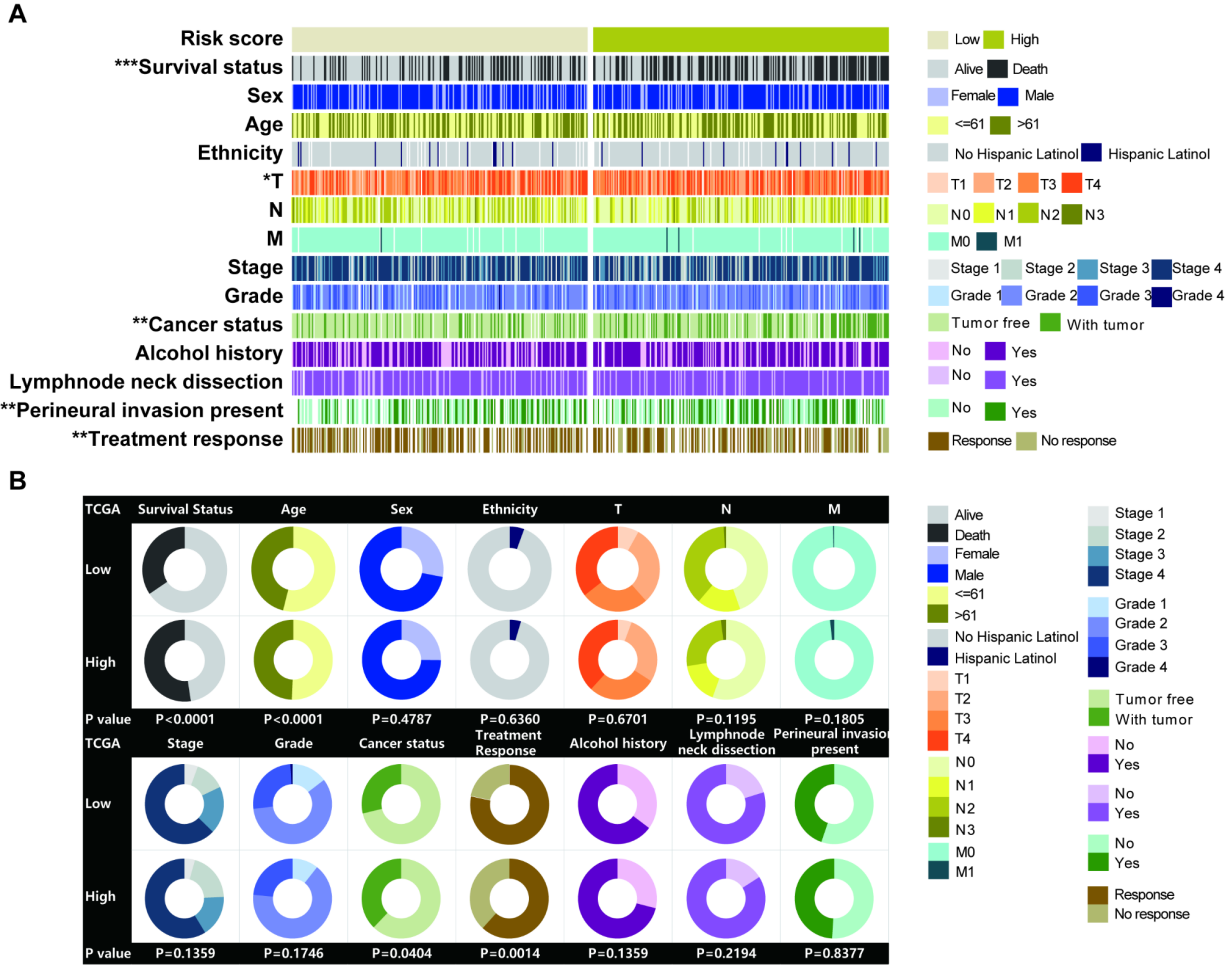
The ERS-related signature is a potential predictor for immunotherapy response in HNSCC. (**A**) The correlation of the ERS-related signature with the expression levels of check-point genes in HNSC from the TCGA. R: Spearman’s correlation coefficient. (**B**) The expression levels of check-point genes among HNSCC samples grouped by risk score in TCGA dataset. Figure S1. Different expression levels of ASNS (**A**) and EXOSC8 (**B**) between normal and tumor samples in HNSCC

Given the association between the ERS-related signature and immune surveillance against cancer cells, we investigated whether the ERS-related signature also correlated with immunotherapy response. Therefore, we first calculated immune check-point scores using the expression levels of immune checkpoint genes (the list of genes shown in Table 2) for each patient by ssGSEA algorithm and measured the association between the risk score and immune check-point score. Strikingly, we observed that the risk score was significantly negatively correlated with the immune check-point score in HNSCC (P < 0.0001, R=-0.2034, Fig. 8A). The expression patterns of immune check-point genes were further compared between the high- and low-risk groups stratified by the ERS-related signature. And the results revealed that patients with high-risk scores exhibited significantly lower expression levels of immune checkpoint genes than those with low-risk score exhibited (P = 0.0002, Fig. 8B).

## Discussion

HNSCC is a multisite malignancy characterized by a low cure rate and easy recurrence [16]. Recent studies have indicated that HNSCC is one of the few malignant tumors that respond well to immunotherapy [6]. Despite the initial enthusiasm, the clinical benefits of immunotherapy in HNSCC patients are overall limited [17]. Therefore, it is important to exploit a biomarker that can predict the response and durability of clinical benefits of immunotherapy to realize better tailor treatments for individual patients. However, biomarkers that can accurately predict prognosis and immunotherapy in HNSCC are still lacking. ERS has been indicated to play a key role in the process of anti-tumor immune response mediation [15]. Targeting ERS-related components may be a promising way of improving the efficacy of cancer immunotherapies [18], suggests potential of ERS for predicting response to cancer immunotherapies. However, there has been no research about ERS as a biomarker for prognosis and cancer immunotherapies in HNSCC.

Therefore, in this work, we first measured the ERS activity in normal and tumor samples using the ssGSEA algorithm and found that the activity of ERS was significantly different between tumor and normal samples of HNSCC. These data hint to us that the ERS activity may be associated with HNSCC. Subsequently, Cox regression analysis was carried out and we mined six ERS-related genes (ASNS, EXOSC6, BAK1, TPP1, EXOSC8, and TATDN2) that exhibited significant prognostic value for OS in HNSCC patients. K-M curves (Log-rank P = 0.0002) and ROC curves (AUC of 0.687) also indicated that these genes had a great value for predicting 5-year OS for HNSCC. Surprisingly, we found that the ERS-related signature could also predict RFS, DSS, and PFS of HNSCC patients, and ROC curves indicated high prediction performance for RFS, DSS, and PFS in HNSCC.

Further correlation analysis of the ERS-related signature with clinical characteristics of HNSCC displayed that the risk of patient mortality rate increased, while the T staging gradually rose and the treatment response rate markedly decreased as the risk score increased, which were in consistence with our previous results that the ERS-related signature is a risk factor for HNSCC prognosis and is associated with grim clinical outcomes.

In this study, we developed and validated an ERS-related prognostic signature (ASNS, EXOSC6, BAK1, TPP1, EXOSC8, and TATDN2) for HNSCC. Asparagine synthetase (ASNS) has been proven to promote cell proliferation and tumor growth in multiple cancers [19, 20]. Moreover, *ASNS* is also associated with cancer metastasis [21]. The researchers indicated that circulating tumor cells could survive insults such as hypoxia and nutrient deprivation as the result of upregulation of factors such as *ATF4, ATF3*, and *ASNS* which were beneficial for cancer cell survival once they detach from the primary tumor and enter the bloodstream. Exosome Component 6 (*EXOSC6*) derived from intracellular proteins has either well-defined or putative roles in breast cancer development and progression [22]. *BAK1* is reported as a prognosis-related gene for HNSCC [23, 24]. Tripeptidyl-peptidase 1 (*TPP1*) is overexpressed in hepatocellular carcinoma (HCC) tissues and significantly correlated with poor prognosis [25]. Moreover, in larynx squamous cell carcinoma, the expression of *TPP1* is elevated, and TPP1 may be a prognostic marker for response to radiotherapy for patients [26]. The oncogenic roles of Exosome Component 8 (EXOSC8) have been confirmed in colorectal carcinoma [27] and prostate cancer [28]. Bin Yu et al. find that the TatD DNase domain containing 2 (TATDN2) is related to early recurrence and has the potential for predicting the prognosis of hepatocellular carcinoma [29]. These previous data are consistence with our results that the six ERS-related genes are related to the prognosis of HNSCC, and have predicted potential for the survival of patients with HNSCC.

Tao Qu et al. developed a prognostic signature consisting of 10 ERS-related LncRNAs for HNSCC [30], which contained huge numbers of RNA, resulting in higher costs and great expenses for clinical applications. In contrast, our signature has a lower cost and is more applicable. Moreover, Tao Qu`s signature was proven to predict OS for HNSCC patients, while our prognostic signature not only can predict OS but also showed predictive power for RFS, DSS, and PFS.

To further unveil the potential mechanism of the correlation of the ERS-related signature with the prognosis of HNSCC, GSEA analysis was employed in this work. The results showed that negative regulation of ubiquitin-dependent protein catabolic process was upregulated in low-risk score groups, suggesting that our signature associated with ERS was robust. Surprisingly, we also found that some immune-associated pathways upregulated in low-risk score groups in HNSCC, such as response to interleukin 4 and B cell receptor signaling pathway, implying our ERS-associated signature might be associated with immune surveillance against cancer cells. Previous research has proved that ERS usually plays a vital role in mediating immune surveillance against cancer cells [15]. Results of GSEA analysis also show that the ERS-related signature is associated with tumor immunity in HNSCC.

Therefore, to further illustrate the potential mechanism of the correlation between the ERS-related signature and immune surveillance against cancer cells, we applied the CIBERSORT algorithm to TCGA dataset. The results indicated that the infiltration of naive B cells, CD8 + T cells, follicular helper T cells, and plasma cells were significantly higher in low-risk groups than in high-risk groups. However, macrophages and activated mast cells were remarkably elevated in high-risk groups than in low-risk groups. The functions of, and interactions between, the innate and adaptive immune systems are vital for anti-tumor immunity. Naive B cells undergo robust proliferation and differentiation that can result in the production of anti-tumor factors such as memory B cells through appropriate stimulation [31, 32]. CD8 + T cells are the most powerful effectors in the anti-tumor immune response and form the backbone of current successful cancer immunotherapies [33]. However, mast cells are proven to support pro-cancerous immune cell infiltrations which induced tumor progression [34, 35]. This research is consistent with our results that the high-risk score is related to low anti-tumor immunity and results in a poor outcome clinical in HNSCC.

Given the association between the ERS-related signature and immune surveillance against cancer cells, we further investigate the correlation between our ERS-related signature and immunotherapy response. Surprisingly, we found that the risk score was significantly negatively correlated with the immune check-point score in HNSCC. Moreover, patients with high-risk scores exhibited significantly lower expression levels of immune checkpoint genes compared with those in the low groups. These results suggest that our ERS-related signature is correlated with immunotherapy response and can be a potential biomarker for immunotherapy response. Of course, the association between the ERS-related signature and immunotherapy response revealed in this study needs to be validated in clinical.

## Conclusion

In conclusion, in this study, we mine an independent and accurate ERS-related signature that can predict OS, RFS, DFS, and PFS of HNSCC patients. GSEA analysis unveils that the ERS-related signature is strongly associated with tumor immunity. Moreover, the infiltration of naive B cells and CD8 + T cells are significantly elevated in patients with low-risk scores compared to those with the high-risk score. By contrast, macrophages and activated mast cells are remarkably enhanced in high-risk groups compared to those in low-risk groups. Furthermore, we find that the ERS-related signature can also be a potential biomarker for immunotherapy response in HNSCC. To the best of our knowledge, this is the first study to analyze the predictive potential of ERS-related genes for prognosis and immunotherapy response in HNSCC, highlighting the impact of ERS-related genes on immune response, potentially enabling more precise and personalized immunotherapy response in the future.

## Electronic supplementary material

Below is the link to the electronic supplementary material.

Supplementary Material 1

Supplementary Material 2

## Data Availability

The original TCGA data that support the findings of our study are available in the NCI GDC Data portal repository at the following URL: https://portal.gdc.cancer.gov/repository. Gene Expression Omnibus (GEO, https://www.ncbi.nlm.nih.gov/geo/) data were from NCBI GEO (accession numbers: GSE65858).

## References

[1] Bray F (2018). Global cancer statistics 2018: GLOBOCAN estimates of incidence and mortality worldwide for 36 cancers in 185 countries. Cancer J Clin.

[2] Peitzsch C, Nathansen J, Schniewind SI, Schwarz F, Dubrovska A. Cancer Stem cells in Head and Neck squamous cell carcinoma: identification, characterization and clinical implications. Cancers (Basel). 2019;11. 10.3390/cancers11050616. 10.3390/cancers11050616 PMC6562868 31052565

[3] Yan B (2013). Unraveling regulatory programs for NF-kappaB, p53 and microRNAs in head and neck squamous cell carcinoma. PLoS ONE.

[4] Farkona S, Diamandis EP, Blasutig IM (2016). Cancer immunotherapy: the beginning of the end of cancer?. BMC Med.

[5] Ferris RL (2015). Immunology and Immunotherapy of Head and Neck Cancer. J Clin oncology: official J Am Soc Clin Oncol.

[6] Cohen EEW (2019). The Society for Immunotherapy of Cancer consensus statement on immunotherapy for the treatment of squamous cell carcinoma of the head and neck (HNSCC). J Immunother Cancer.

[7] Hegde PS, Chen DS (2020). Top 10 Challenges in Cancer Immunotherapy. Immunity.

[8] Bertolotti A, Zhang Y, Hendershot LM, Harding HP, Ron D (2000). Dynamic interaction of BiP and ER stress transducers in the unfolded-protein response. Nat Cell Biol.

[9] Oakes SA, Papa FR (2015). The role of endoplasmic reticulum stress in human pathology. Annu Rev Pathol.

[10] Chen W (2021). Downregulation of ceramide synthase 1 promotes oral cancer through endoplasmic reticulum stress. Int J Oral Sci.

[11] Afonyushkin T (2010). Oxidized phospholipids regulate expression of ATF4 and VEGF in endothelial cells via NRF2-dependent mechanism: novel point of convergence between electrophilic and unfolded protein stress pathways. Arterioscler Thromb Vasc Biol.

[12] Hetz C, Martinon F, Rodriguez D, Glimcher LH (2011). The unfolded protein response: integrating stress signals through the stress sensor IRE1α. Physiol Rev.

[13] Hänzelmann S, Castelo R, Guinney J. GSVA: gene set variation analysis for microarray and RNA-seq data. BMC Bioinformatics. 2013;14. 10.1186/1471-2105-14-7. 10.1186/1471-2105-14-7 PMC3618321 23323831

[14] Newman AM (2015). Robust enumeration of cell subsets from tissue expression profiles. Nat Methods.

[15] Cubillos-Ruiz JR, Bettigole SE, Glimcher LH (2017). Tumorigenic and immunosuppressive Effects of endoplasmic reticulum stress in Cancer. Cell.

[16] Xiong Y (2021). Prognostic value of lipid metabolism-related genes in head and neck squamous cell carcinoma. Immun Inflamm Dis.

[17] Ferris R, Gillison ML. Nivolumab for squamous-cell Cancer of Head and Neck. N Engl J Med. 2017;376. 10.1056/NEJMc1615565. 10.1056/NEJMc1615565 28177863

[18] Mohamed E, Cao Y, Rodriguez PC (2017). Endoplasmic reticulum stress regulates tumor growth and anti-tumor immunity: a promising opportunity for cancer immunotherapy. Cancer Immunol immunotherapy: CII.

[19] Yu Q (2016). Knockdown of asparagine synthetase (ASNS) suppresses cell proliferation and inhibits tumor growth in gastric cancer cells. Scand J Gastroenterol.

[20] Pathria G (2019). Translational reprogramming marks adaptation to asparagine restriction in cancer. Nat Cell Biol.

[21] Ameri K (2010). Circulating tumour cells demonstrate an altered response to hypoxia and an aggressive phenotype. British J cancer.

[22] Hawkins OE (2008). Identification of breast cancer peptide epitopes presented by HLA-A*0201. J Proteome Res.

[23] Jin Y, Qin X (2020). Development of a prognostic signature based on autophagy-related genes for Head and Neck squamous cell carcinoma. Arch Med Res.

[24] Zhu L (2020). The identification of autophagy-related genes in the prognosis of oral squamous cell carcinoma. Oral Dis.

[25] Zhao Y (2017). The transcription factor RFX5 is a transcriptional activator of the TPP1 gene in hepatocellular carcinoma. Oncol Rep.

[26] Tang T (2009). Increased expression of telomere-related proteins correlates with resistance to radiation in human laryngeal cancer cell lines. Oncol Rep.

[27] Cui K, Liu C, Li X, Zhang Q, Li Y (2020). Comprehensive characterization of the rRNA metabolism-related genes in human cancer. Oncogene.

[28] Ngollo M, et al. Global analysis of H3K27me3 as an epigenetic marker in prostate cancer progression. BMC Cancer. 2017;17. 10.1186/s12885-017-3256-y. 10.1186/s12885-017-3256-y PMC5388998 28403887

[29] Yu B, Liang H, Ye Q, Wang Y (2021). Establishment of a genomic-clinicopathologic Nomogram for Predicting Early recurrence of Hepatocellular Carcinoma after R0 resection. J Gastrointest Surg.

[30] Qu T (2022). Prognostic signature of endoplasmic reticulum stress-related long noncoding RNAs in head and neck squamous cell carcinoma: Association with somatic mutation and tumor immune microenvironment. J Dent Sci.

[31] Steach HR et al. Cross-Reactivity with Self-Antigen Tunes the Functional Potential of Naive B Cells Specific for Foreign Antigens. Journal of immunology (Baltimore, Md: 1950) 2020;204:498–509, 10.4049/jimmunol.1900799. 10.4049/jimmunol.1900799 PMC6981075 31882518

[32] Dahl M, Kristensen LS, Grønbæk K. Long non-coding RNAs Guide the Fine-Tuning of Gene Regulation in B-Cell Development and Malignancy. Int J Mol Sci. 2018;19. 10.3390/ijms19092475. 10.3390/ijms19092475 PMC6165225 30134619

[33] Raskov H, Orhan A, Christensen JP, Gögenur I (2021). Cytotoxic CD8 T cells in cancer and cancer immunotherapy. Br J Cancer.

[34] Okano M, et al. Triple-negative breast Cancer with high levels of annexin A1 expression is Associated with mast cell infiltration, inflammation, and Angiogenesis. Int J Mol Sci. 2019;20. 10.3390/ijms20174197. 10.3390/ijms20174197 PMC6747082 31461932

[35] Gorzalczany Y, Akiva E, Klein O, Merimsky O, Sagi-Eisenberg R (2017). Mast cells are directly activated by contact with cancer cells by a mechanism involving autocrine formation of adenosine and autocrine/paracrine signaling of the adenosine A3 receptor. Cancer Lett.

