# Supplementary figures and images for: Development of an endoplasmic reticulum stress-related signature with potential implications in prognosis and immunotherapy in head and neck squamous cell carcinoma
Source: Diagn Pathol. 2023 Apr 22;18:51. doi: 10.1186/s13000-023-01338-4 (PMC10122290; doi:10.1186/s13000-023-01338-4)

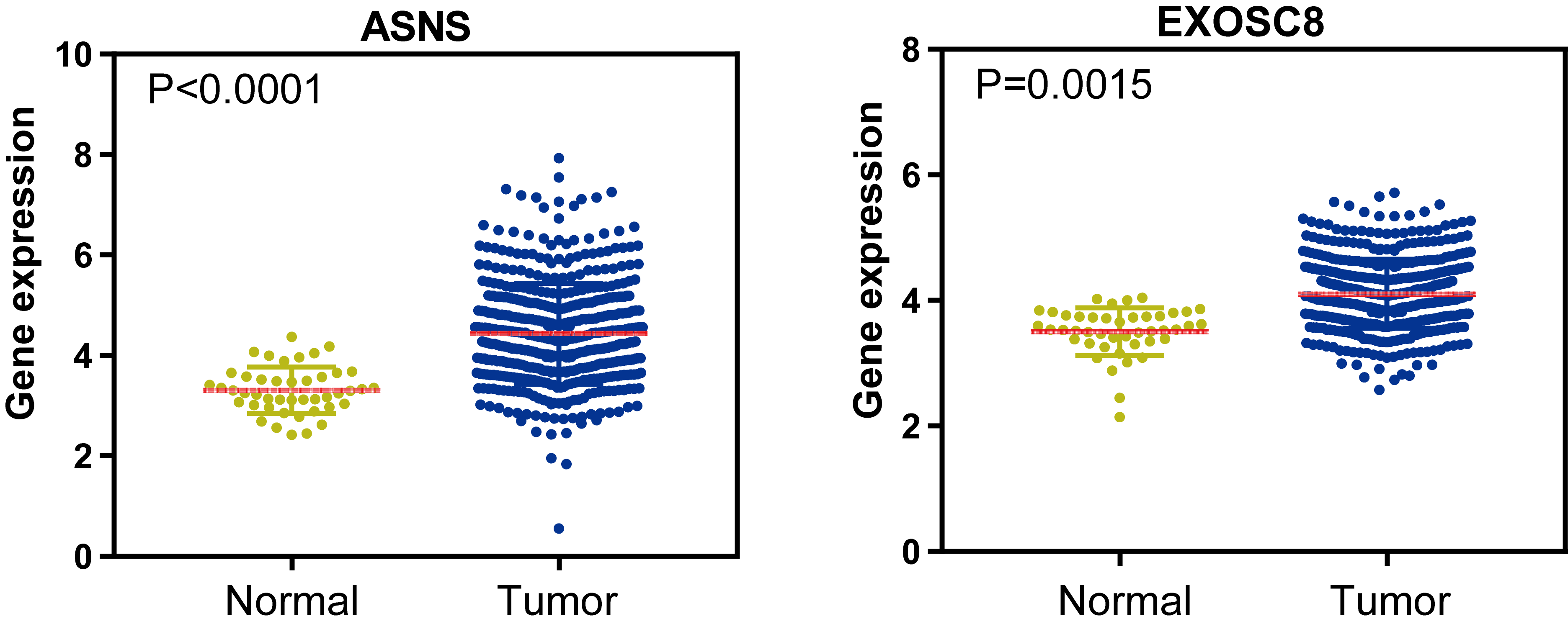

Supplement: Supplementary file 1 — Supplementary Material 1 [file 13000_2023_1338_MOESM1_ESM.png]
